# Sonic hedgehog signaling is negatively regulated in reactive astrocytes after forebrain stab injury

**DOI:** 10.1038/s41598-018-37555-x

**Published:** 2019-01-24

**Authors:** R. Vivian Allahyari, K. Lyles Clark, Katherine A. Shepard, A. Denise R. Garcia

**Affiliations:** 1 Departments of Biology and Neurobiology and Anatomy, Drexel University, Philadelphia, PA 19104 USA; 2 Present Address: Mahoney Institute for Neurosciences, Perelman School of Medicine, University of Pennsylvania, Philadelphia, Pennsylvania; and Department of Anesthesiology and Critical Care Medicine, Children’s Hospital of Philadelphia, Philadelphia, Pennsylvania USA

## Abstract

Following injury to the central nervous system, astrocytes perform critical and complex functions that both promote and antagonize neural repair. Understanding the molecular signaling pathways that coordinate their diverse functional properties is key to developing effective therapeutic strategies. In the healthy, adult CNS, Sonic hedgehog (Shh) signaling is active in mature, differentiated astrocytes. Shh has been shown to undergo injury-induced upregulation and promote neural repair. Here, we investigated whether Shh signaling mediates astrocyte response to injury. Surprisingly, we found that following an acute, focal injury, reactive astrocytes exhibit a pronounced reduction in Shh activity in a spatiotemporally-defined manner. Shh signaling is lost in reactive astrocytes at the lesion site, but persists in mild to moderately reactive astrocytes in distal tissues. Nevertheless, local pharmacological activation of the Shh pathway in astrocytes mitigates inflammation, consistent with a neuroprotective role for Shh signaling after injury. Interestingly, we find that Shh signaling is restored to baseline levels two weeks after injury, a time during which acute inflammation has largely subsided and lesions have matured. Taken together, these data suggest that endogenous Shh signaling in astrocytes is dynamically regulated in a context dependent manner. In addition, exogenous activation of the Shh pathway promotes neuroprotection mediated by reactive astrocytes.

## Introduction

Following injury to the central nervous system (CNS), the molecular signaling pathway, Sonic hedgehog (Shh), has been shown to exert neuroprotective effects on multiple cell types. SHH promotes proliferation of oligodendrocyte progenitor cells as well as adult neural stem and progenitor cells in various injury models including spinal cord injury, stroke, and cortical stab injuries^1–7^. In addition, SHH acts on endothelial cells to promote blood brain barrier integrity and modulates neuroinflammatory signaling, mitigating inflammation in the CNS^8,9^. However, in the healthy brain, canonical Shh signaling occurs predominantly in astrocytes, which play key roles in both neuroprotective and neuroinflammatory actions in the injured CNS^10^. The role of Shh signaling in mediating the response of astrocytes to injury is not well understood.

Transduction of Shh signaling occurs through the family of GLI transcription factors. Initiation of pathway activity occurs upon binding of SHH to its receptor, Patched (PTC), removing inhibition of Smoothened (SMO), an obligatory component of all Shh signaling^11^. Activation of SMO triggers posttranslational processing of GLI proteins that promote transcription of SHH target genes, including *Gli1*, which serves as a reliable readout of active Shh signaling^12–14^. We previously reported that in the healthy, mature forebrain, astrocytes are the predominant cells expressing *Gli1*^15^. Astrocytes are key components of the neural response to injury and facilitate cellular processes that both promote and inhibit neural repair^10^. It has been shown that reactive astrocytes exhibit neural stem cell properties *in vitro* in response to SHH^7^. Whether Shh signaling mediates reactive astrogliosis *in vivo* remains poorly understood.

In this study, we show a pronounced reduction in *Gli1* expression in reactive astrocytes following a unilateral and invasive focal injury to the forebrain, suggesting that Shh signaling declines after injury. Interestingly, loss of Shh signaling occurs in a temporally and spatially defined manner, with prominent loss of Shh activity in cells and tissues proximal to the injury, while Shh signaling persists in cells distal from the lesion. Shh activity is restored to baseline levels by 14 days post injury (dpi), when proliferation of reactive astrocytes is largely complete and the glial scar matures^16^. Despite the lack of available SHH, local pharmacological activation of the Shh signaling pathway during the acute injury phase limits leukocyte migration in parenchymal tissues adjacent to the lesion. This effect is mediated by SMO-dependent signaling in reactive astrocytes, suggesting that despite the absence of SHH, exogenous activation of the pathway mitigates inflammation. Taken together, these data suggest that Shh signaling in astrocytes is negatively regulated during acute reactive gliosis but is restored as inflammation and gliosis begin to subside and the injury response approaches resolution. This suggests that astrocytic Shh signaling undergoes dynamic spatiotemporal regulation in a context-dependent manner. Finally, these data further suggest that despite a loss of available SHH after injury, exogenous interventions that promote Shh activity can promote neuroprotection.

## Results

We began by investigating the phenotypic characteristics of reactive Gli1 astrocytes following a forebrain stab injury, a well-established model of acute, focal trauma that reliably and robustly triggers proliferation of reactive astrocytes and glial scar formation^17,18^. We used an inducible recombination strategy to selectively mark and identify cells expressing *Gli1*, enabling us to monitor their behavior in response to a subsequent forebrain stab. Transgenic mice in which CreER is targeted to the *Gli1* locus (*Gli1*^*CreER*/+^)^12^ were crossed with the Rosa26tdTomato reporter line (*Rosa26*^*flSTOPfltdTomato*^, line Ai14)^19^ to generate *Gli1*^*CreER*/+^;*R26*^*tdT*/*tdT*^ mice. In a previous study using *Gli1*^*CreER*/+^;*R26*^*lacZ*/*lacZ*^ mice, we found that regulation of reporter protein from the *Rosa26* locus is weaker in astrocytes than neurons, and requires at least 4–6 weeks to observe complete expression of βGal^15^. For these studies, we found no difference in the number, distribution, or fluorescence intensity of tomato expressing cells between 2 and 4 weeks following tamoxifen (Supplementary Fig. S1). We therefore used a minimum of 2 weeks after tamoxifen as our standard protocol for this study, except where otherwise noted.

**Figure 1:**
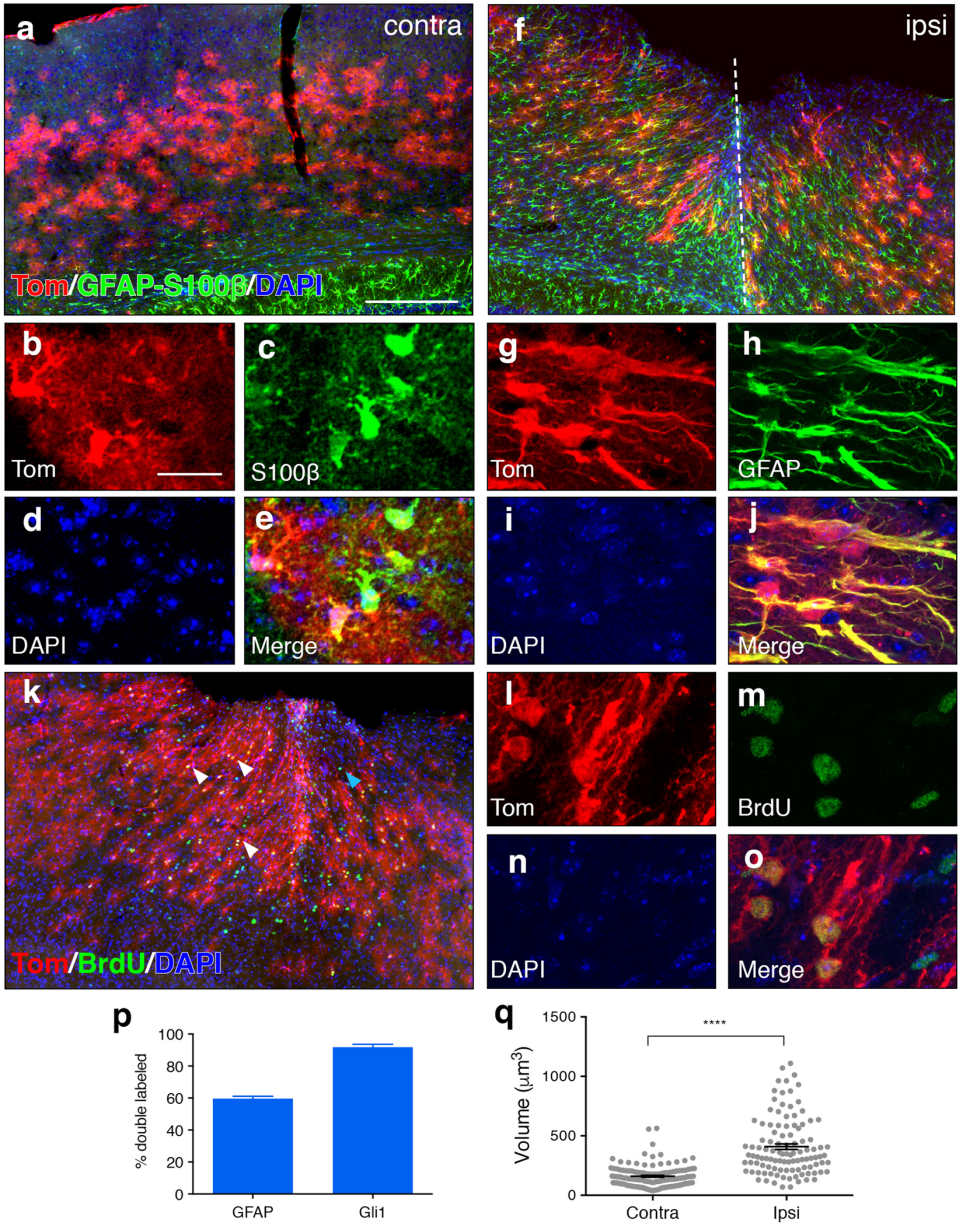
Gli1 astrocytes marked before injury exhibit features of reactive astrocytes. (**a**,**f**) Low power micrographs from the contralateral (**a**) and ipsilateral (**f**) cortex of an adult *Gli1*^*CreER*/+^;*R26*^*tdTomato*/*tdTomato*^ mouse that received tamoxifen 2 weeks before injury. Fluorescence immunohistochemistry for GFAP-S100β (green) shows that Gli1 astrocytes (red) at the lesion site upregulate GFAP and extend long radial processes towards the blade track (dashed line). (**b**–**e**,**g**–**j**) Maximum projection images from confocal z-stacks showing colocalization of marked Gli1 astrocytes (red, **b, g**) and S100β or GFAP (green, **c**,**h**). Tissues counterstained with DAPI (blue, **d**,**i)**. Merged image shown in (**e**,**j**). (**k**) Low power micrograph of tomato (red) and BrdU (green) labeled cells at the lesion site showing marked astrocytes at the lesion site proliferating. (**l**–**o**) Maximum projection images from a confocal stack showing colocalization of tomato (red, l) and BrdU (green, m). Tissues counterstained with DAPI (blue, n). Merged image shown in (**o**). (**p**) Single cell analysis of the proportion of GFAP and Gli1 cells that are colabeled (*n* = 1,527 and *n* = 990 cells, respectively, from 3 animals). Error bars represent mean ± SEM. (**q**) Stereological estimates of cell body volume of marked cells in contralateral and ipsilateral hemispheres. Data points represent individual cells, bars represent mean ± SEM. Statistical signficance was assessed by unpaired Student’s t-test (*p* < 0.0001, *n* = 131 and *n* = 108 cells, respectively from 4 animals). Scale bars, 250 µm (**a**,**f**,**k**), 25 µm (**b**–**e**, **g**–**j**, **l**–**o**).

We administered tamoxifen by oral gavage to adult *Gli1*^*CreER*/+^;*R26*^*tdT*/*tdT*^ mice and performed a unilateral stab injury in the right hemisphere 2 weeks later. We evaluated marked cells for several key hallmarks of reactive astrocytes, including upregulation of the intermediate filament, glial fibrillary acidic protein (GFAP), and cellular hypertrophy. At 7 days post injury (dpi), marked, tomato-expressing cells in the ipsilateral cortex show robust staining for GFAP (Fig. 1). Single cell colocalization analysis of marked cells in the ipsilateral cortex showed that 92% of marked cells were co-labeled with GFAP (Fig. 1). In contrast, we observed little to no labeling of GFAP in the cortex of the intact, contralateral hemisphere (Fig. 1), consistent with the fact that GFAP expression is low in the healthy, uninjured cortex^20^. We nevertheless identified marked cells in the contralateral hemisphere as astrocytes by colocalization with S100β (Fig. 1), consistent with our previous observation that 98% of marked cells in the mature, uninjured cortex correspond to astrocytes^15^. In the ipsilateral cortex, double labeled cells exhibited elongated morphologies, with radially oriented processes that were in stark contrast to the bushy appearance and stellate morphologies of marked cells in the contralateral hemisphere (Fig. 1). These elongated morphologies were most pronounced at the lesion site (defined as the blade track) and within 500 μm mediolaterally from the lesion, collectively referred to as the lesion area. In addition, marked cells in the ipsilateral cortex showed dramatic cellular hypertrophy. We performed stereological estimates of the volume of individual cell bodies marked in the ipsilateral and contralateral hemispheres using a nucleator probe. Marked cells in the ipsilateral cortex showed a significant increase in cell volume compared to cells in the contralateral hemisphere (408.8 ± 23.34 μm^3^ and 160.1 ± 7.738 μm^3^, respectively, Fig. 1), indicating cellular hypertrophy consistent with reactive gliosis. We further evaluated the fraction of GFAP-labeled reactive astrocytes in the lesion area that were marked with tomato. GFAP staining is typically absent in cell bodies, localizing primarily to astrocytic processes. To definitively identify GFAP-stained cells, we identified GFAP processes with clear nuclear staining for DAPI. We found that 60% of GFAP labeled cells in the lesion area were marked with tomato labeling (Fig. 1), consistent with our previous observation that only a fraction of cortical astrocytes express *Gli1*^15^. These data show that Gli1 cells exhibit phenotypic characteristics consistent with reactive astrocytes, and that these cells comprise a substantial fraction of the reactive astrocyte population that is triggered by cortical stab wound.

It has been reported that NG2 cells adopt phenotypic characteristics of reactive astrocytes in response to Shh activity^5^. We examined whether marked cells co-label with NG2 in the contralateral and ipsilateral hemispheres of injured mice. Single cell analysis showed that few marked cells co-labeled with NG2 in both hemispheres (0.5% and 0.4% in contralateral and ipsilateral hemispheres, respectively, Supplemental Fig. S2), indicating that these cells do not contribute substantially to our marked population of Gli1 cells, and consistent with our previous observation that the vast majority of marked cells in the mature, uninjured brain correspond to astrocytes^15^.

Reactive astrogliosis is not all or none and encompasses distinct gene expression patterns and cellular behaviors that vary with respect to injury type and severity^16,21–24^. The forebrain stab injury model triggers severe astrogliosis that includes proliferation of reactive astrocytes and glial scar formation^18,25^. SHH plays a critical role in regulating proliferation of neural stem and progenitor cells in the developing and mature nervous system^26–30^. In addition, pharmacological approaches that promote or antagonize Shh activity have been shown to increase and decrease proliferation, respectively, following cortical stab wound^1,7^. We therefore hypothesized that proliferating, reactive astrocytes are comprised of Gli1 astrocytes and investigated whether marked cells proliferate in response to injury. We administered the thymidine analog, BrdU, by daily i.p. injection over days 3–6 following injury, the peak of reactive astrocyte proliferation^16^, and examined tissues at 7 dpi. Single-cell colocalization analysis showed that 59% of marked cells in the lesion area incorporated BrdU (*n* = 445 cells from 3 animals, Fig. 1). Taken together, these data show that Gli1 astrocytes marked before injury exhibit several key phenotypic and physiological features of reactive astrocytes and suggest that these cells participate in the neural response to injury.

### Shh activity is lost in reactive astrocytes proximal to the injury

**Figure 2:**
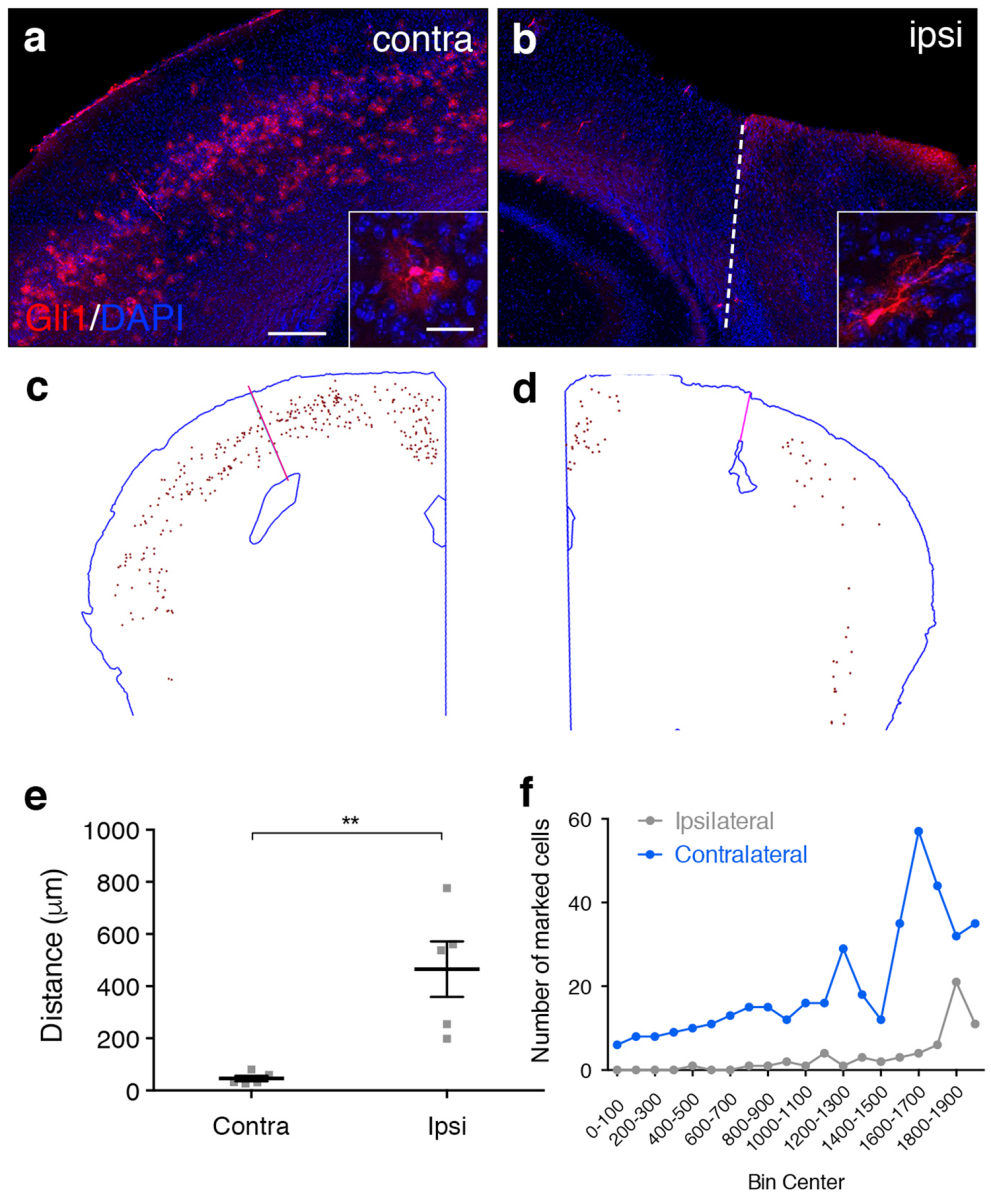
*Gli1* expression is lost in the lesion area. (**a**,**b**) Tomato expression (red) in the contralateral (**a**) and ipsilateral (**b**) cortex of an adult *Gli1*^*CreER*/+^;*R26*^*tdTom*/*tdTom*^ mouse that received a single dose of tamoxifen on day 3 after injury. Dashed line indicates blade track. Insets show single cells from contralateral and ipsilateral hemispheres. Note the pronounced change in size and morphology of the marked cell in the ipsilateral, compared to the intact, contralateral hemisphere. Scale bar, 250 µm, inset, 50 µm. Counterstained with DAPI (blue). (**c**,**d**) Stereo Investigator tracings showing the distribution of Gli1 astrocytes throughout the contralateral (**c**) and ipsilateral (**d**) hemispheres from animals that received a single dose of tamoxifen at 3 dpi. Pink line denotes the actual or projected blade track. Note the pronounced absence of marked cells in the region immediately surrounding the lesion in the ipsilateral hemisphere, and a relative reduction in the number of marked cells throughout the ipsilateral cortex, relative to the intact, contralateral hemisphere. (**e**) The average distance from the actual or projected blade track to the first marked cell on the ipsilateral or contralateral hemispheres, respectively. Data points represent independent animals, bars represent mean ± SEM. Statistical significance assessed by unpaired Student’s t-test (*p* = 0.0004). (**f**) The number of marked cells throughout the ipsilateral and contralateral hemispheres, relative to their distance from the lesion, plotted in 100 µm bins. Pooled data from 4 animals.

The role of SHH in regulating proliferation of neural precursor cells in the developing and adult CNS is well established^31–34^. Although astrocytes in the healthy brain do not proliferate, reactive astrocytes can undergo proliferation in response to a severe focal insult^18,35^. In order to examine whether Shh signaling plays a role in regulating astrocyte proliferation following stab injury, we used genetic marking of Gli1 astrocytes to identify these cells and subsequently monitor their behavior after injury. Animals received a single dose of tamoxifen on day 3 after injury, when astrocytes exhibit high rates of injury-induced proliferation. Tissues were analyzed at short (4 days) and long term (14 days) time points after tamoxifen (7 and 17 dpi) in order to observe whether the number of marked cells increases over time. In addition, this strategy enabled us to investigate whether individual cells that lack *Gli1* expression in the healthy brain subsequently upregulate *Gli1* in response to injury. Because tamoxifen-mediated recombination selectively marks cells actively expressing *Gli1* and their progeny, cells that upregulate *Gli1* after injury will not be detected in animals receiving tamoxifen before injury. We reasoned that injury-induced upregulation of *Gli1* should result in an increase in the number of marked cells in tissues marked after, compared to before, injury. Tissues were stained with an antibody against red fluorescent protein (RFP) and we examined the number and distribution of marked cells in the lesion area. Unexpectedly, we observed a striking absence of marked cells in the lesion area at both time points (Supplementary Fig. S3). This was in stark contrast to the corresponding region of cortex in the intact contralateral hemisphere, where the number and distribution of Gli1 astrocytes remained indistinguishable from uninjured controls (Supplementary Fig. S3). To rule out the possibility that a single dose of tamoxifen was insufficient to mark Gli1 cells, we administered tamoxifen over 3 days, beginning at 3 dpi, and analyzed tissues two weeks later. Although more marked cells were detected in the lesion area, compared to that observed with a single dose of tamoxifen, the lesion area remained relatively devoid of marked cells, compared to the contralateral hemisphere (Supplementary Fig. S3). In order to examine the distribution of marked cells after injury, we mapped all labeled cells in both hemispheres in sparsely labeled tissues (Fig. 2). We identified and traced the blade track on the ipsilateral hemisphere and subsequently projected the traced lesion onto the contralateral hemisphere. The distance from the lesion or projected lesion to the nearest marked cell was significantly larger on the ipsilateral, compared to the contralateral, hemisphere (465 ± 106.5 μm and 46.08 ± 10.35 μm, respectively, Fig. 2). We quantified the number of cells that were observed within 2 mm mediolaterally from the lesion or projected lesion on the ipsilateral and contralateral hemispheres, respectively. This analysis showed a dramatic reduction in the number of marked cells throughout the ipsilateral compared to contralateral hemisphere, suggesting that loss of *Gli1* extended beyond the lesion area (Fig. 2). Nevertheless, marked cells were observed in ipsilateral tissues distal from the lesion (Fig. 2), suggesting that Shh signaling persists in cells and tissues experiencing less severe inflammation and where the blood brain barrier (BBB) remains intact. Marked cells in both hemispheres showed an astrocytic morphology (Fig. 2), and we did not detect any marked cells with a neuronal or oligodendrocytic morphology, consistent with our previous study^15^. However marked cells displayed dramatic changes in morphology, similar to that observed in tissues marked before injury, suggesting that we were marking a similar population of cells before and after injury. Marked cells exhibited elongated processes, oriented towards the lesion, with hypertrophic cell bodies, consistent with the morphology of reactive astrocytes (Fig. 2). Despite the pronounced reduction in marked cells in the lesion area, the overall distribution remained consistent between both hemispheres (Fig. 2). These data show that injury triggers a dramatic loss of *Gli1* expression within 3 days. This loss of *Gli1* is most prominent in astrocytes proximal to the lesion that are undergoing severe reactive gliosis, whereas *Gli1* expression largely persists in astrocytes distal from the lesion.

A small fraction of marked cells in the ipsilateral lesion area co-labeled with NG2, in animals that received either one or three doses of tamoxifen. However these cells were largely found along vasculature, and exhibited a morphology consistent with pericytes (Supplementary Fig. 2). This is consistent with a recent report that a small fraction of pericytes express NG2^5^. Interestingly, we observed a modest increase in these marked, NG2-expressing pericytes in tissues marked after, compared to before, injury (10.3% and 0.4%, marked after and before, respectively, Supplementary Fig. 2). This was observed in the ipsilateral hemisphere only. This suggests that NG2-expressing pericytes upregulate *Gli1* in injured tissues, though the total number of cells in which this was observed was exceedingly low (2 cells and 16 cells before and after injury, respectively, from 2 animals). Analysis of the fraction of NG2 cells colabeled with tomato in tissues marked after, compared to before, injury showed a small increase (2.2% and 0.3% marked after and before, respectively; Supplementary Fig. 2). However these cells exhibited a pericyte-like morphology, and we found no evidence for upregulation of *Gli1* in NG2 labeled cells exhibiting an NG2-like morphology. This suggests that injury does not induce upregulation of *Gli1* in NG2 cells. Taken together, these data suggest that, *Gli1* expression is dramatically reduced in the lesion area following injury. Moreover, these data show that, as in the healthy adult brain, canonical Shh signaling occurs predominantly in astrocytes, before and after injury.

**Figure 3:**
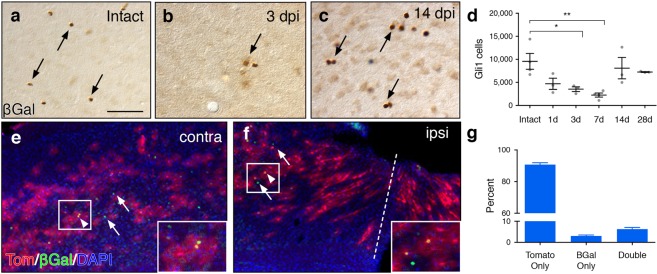
*Gli1* expression is downregulated in the lesion area. (**a**–**c**) Brightfield immunohistochemistry for βGal in *Gli1*^*nLacZ*/+^ mice showing the number of Gli1 cells in the intact cortex (**a**), and at 3 (**b**) and 14 (**c**) days after injury. Scale bar, 50 µm. (**d**) Stereological quantification of the number of Gli1 cells in the lesion area. Data points represent individual animals, bars represent mean ± SEM. Statistical significance was assessed by one-way ANOVA, post-hoc Tukey’s multiple comparisons test (*p = 0.047, **p = 0.007). (**e**,**f**) Distribution of tomato (red) and βGal (green) labeled cells in the contralateral (**e**) and ipsilateral (**f**) hemispheres of a *Gli1*^*CreER*/*nLacZ*^;*R26*^*tdTom*/+^ mouse at 7 dpi. Tissues counterstained with DAPI (blue). A small fraction of βGal labeled cells do not co-express tomato (arrows). Double-labeled cells indicated by arrowheads. (**g**) The fraction of single and double labeled cells in the lesion area of the ipsilateral hemisphere (*n* = 1,464 cells analyzed from 4 animals). Note that the vast majority of marked cells in the lesion area do not co-express βGal (tomato only), reflecting the absence of βGal proximal to the lesion. Bars represent mean ± SEM.

To rule out failure of Cre-mediated recombination due to unhealthy or dying cells, we examined a second, independent transgenic line in which a reporter gene, *lacZ*, is targeted to the *Gli1* locus^13^. In *Gli1*^*nlacZ*/+^ mice, expression of nuclear βGal reporter protein is a direct and reliable readout of transcriptional activation of *Gli1*^13^. In addition, these mice enabled us to examine the time course over which *Gli1* expression is lost. We performed stab injury in adult *Gli1*^*nlacZ*/+^ mice and analyzed βGal staining at various time points after injury. Stereological quantification of the number of βGal cells in the lesion area showed a 51% reduction in the number of Gli1 cells as early as 1 dpi in injured tissues compared to intact controls (4701 ± 1202 and 9568 ± 1703, respectively, Fig. 3). The number of Gli1 cells continues to decline such that, by 7 dpi, there are only 2266 ± 437.2 cells in the lesion area, a 76% reduction in the number of Gli1 cells compared to intact controls.

To rule out the possibility that the reduction in the number of Gli1 reactive astrocytes in the lesion area is a result of cell death, we generated double reporter mice expressing CreER and nuclear lacZ from the *Gli1* locus (*Gli1*^*CreER*/*nlacZ*+^;*R26*^*tdT*/+^). If the apparent loss of *Gli1* expression is due to cell death, we would expect to observe an absence of marked cells in the lesion area. We administered tamoxifen 2 weeks before injury and examined the distribution of marked cells and βGal cells in the lesion area at 7 dpi. Consistent with our previous data, marked cells were readily observed throughout the lesion area. These cells exhibited elongated morphologies, with polarized processes oriented towards the lesion (Fig. 3). In contrast, few βGal cells were observed in the lesion area (Fig. 3). Colocalization analysis of tomato and βGal in the lesion area revealed that only 6.25% of all cells analyzed were double labeled. The vast majority (90.74%) of cells analyzed were marked with tomato but were not co-labeled with βGal, reflecting the absence of cells actively expressing *Gli1* in this region. These data suggest that Gli1 astrocytes marked by tamoxifen before injury subsequently lose *Gli1* expression. A small fraction of cells labeled with βGal did not colabel with tomato (3%), reflecting cells that failed to undergo tamoxifen-mediated recombination. Alternatively, these cells may reflect an upregulation of *Gli1* in reactive astrocytes. However, our previous experiment in which we administered tamoxifen after injury failed to mark any cells in the lesion area, arguing against this possibility. Taken together, these data suggest that Shh signaling is lost, not upregulated, following an acute, focal injury. Loss of Shh activity is most prominent in reactive astrocytes adjacent to the insult but is mitigated in distal tissues, indicating that injury-induced loss of Shh activity occurs along a spatially-defined gradient.

**Figure 4:**
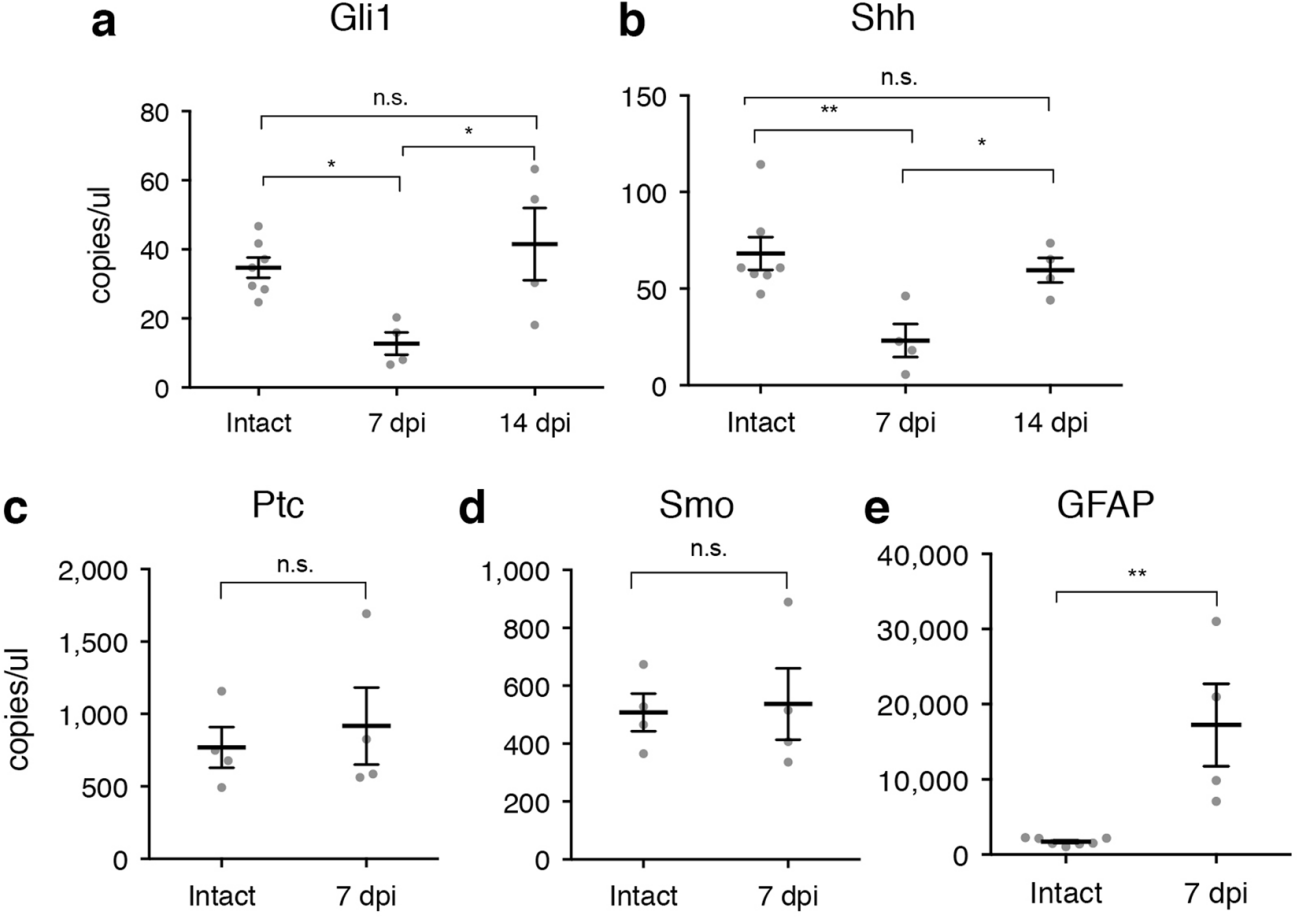
Transcriptional loss of *Shh* and *Gli1*. Absolute quantification of transcripts of multiple components of the Shh signaling pathway (**a**,**d**) and GFAP (**e**) from the cortex of intact or injured mice at 7 and 14 dpi. Data points represent independent animals, bars represent mean ± SEM. Statistical significance was assessed by one-way ANOVA, with post-hoc Tukey’s multiple comparisons test (**a**,**b**), or unpaired Student’s t-test (**c**–**e**); *p < 0.05, **p < 0.01.

Because our transgenic mouse models rely on indirect readouts of GLI1 through analysis of reporter proteins, we directly interrogated *Gli1* levels by measuring transcript copy number. We isolated the cortex, removing subcortical structures including underlying white matter, and microdissected cortical tissues including the lesion area and tissues within a 1500 μm radius from the blade track. We isolated RNA and quantified the absolute number of transcripts in injured and intact tissues at 7 dpi by Droplet Digital^TM^ PCR (ddPCR). We first quantified the number of *Gfap* transcripts in the injured vs. intact hemisphere. Consistent with our histological results, we found a 10-fold increase in *Gfap* transcripts in the injured (17239 ± 5489 copies/μl), compared to intact cortex (1721 ± 177.3 copies/μl, Fig. 4). We next quantified *Gli1* transcripts and observed a significant reduction in the injured (12.71 ± 3.24 copies/μl) compared to intact cortex (34.7 ± 2.96 copies/μl, Fig. 4). The loss of *Gli1* could reflect a reduction in the amount of SHH available following an acute injury. Alternatively, *Gli1* loss could result from a failure of reactive astrocytes to effectively transduce Shh signaling. To distinguish between these possibilities, we quantified the number of *Shh* transcripts at 7 dpi. We found a significant reduction in the absolute number of *Shh* transcripts in the lesioned cortex (23.12 ± 8.514 copies/μl) compared to intact control (68.2 ± 8.504 copies/μl, one-way ANOVA, p = 0.0084), suggesting a reduction in the availability of SHH following an injury (Fig. 4).

Because transcript abundance does not necessarily reflect protein levels, and to rule out the possibility that SHH protein may be available from non-neural sources that invade neural tissues following a stab injury, we measured transcriptional activation of an additional target of the Shh pathway. *Ptc* is itself a transcriptional target of Shh signaling and of GLI1, and undergoes upregulation in response to high levels of SHH^36,37^. PTC serves as a negative regulator of Shh signaling, repressing its own transcription, and maintaining a baseline level of *Ptc* transcript in the absence of SHH^36,38,39^. Quantification of *Ptc* transcripts in intact and injured cortex showed no difference in injured (916.7 ± 265.4 copies/μl), compared to intact tissues (769.2 ±140.6 copies/μl, Fig. 4). This is consistent with a reduction in GLI1 and argues against the possibility of SHH protein from any neural or non-neural sources. Finally, we quantified *Smo* in the intact and 7 dpi cortex and found no difference the number of transcripts of this obligatory component of the Shh signaling cascade (Fig. 4), suggesting that the reduction in transcriptional activation of *Gli1* is a result of lower SHH availability, and not a loss of necessary components to transduce Shh signaling. Taken together, these data demonstrate that following an acute, focal injury, Shh signaling is lost in injured tissues proximal to the lesion.

### Shh signaling is restored at 2 weeks after injury

Our data show that Shh signaling is downregulated in reactive astrocytes during the first week after a forebrain stab injury. Whether this reflects a permanent change in astrocyte phenotype or whether Shh activity returns is unknown. To address this, we performed forebrain stab injury on *Gli1*^*nlacZ*/+^ mice, and quantified the number of βGal cells in the lesion area at 14 and 28 dpi. At 14 dpi, there were 8092 ± 2324 βGal-labeled cells in the lesion area. Notably, this number was not significantly different from intact tissues (9568 ± 1703, Fig. 3), suggesting that Shh activity returns to baseline levels. At 28 dpi, there were 7258 ± 76.04 βGal-labeled cells in the lesion area (Fig. 3), suggesting that the restoration of Shh signaling is persistent. We further quantified the number of *Gli1* transcripts at 14 dpi, and found that injured animals at this time point showed no difference in copy number compared to intact control tissues (41.52 ± 10.47 copies/μl, Fig. 4). Similarly, the number of *Shh* transcripts at 14 dpi (59.52 ± 6.377 copies/μl, Fig. 4) showed no difference compared to control tissues. Taken together, these data indicate that the loss of Shh signaling in injured tissues is temporary and is restored to baseline levels by 14 days after the initial insult.

The restoration of Shh activity to the lesion area suggests that Shh signaling may play a role in the chronic phase of injury. During chronic injury, tissues undergo resolution of BBB damage and glial scars mature^16^. These critical cellular events promote the long-term resolution of injury. To investigate whether permanent loss of Shh signaling impairs neural repair processes during chronic injury, we used a conditional knock out (CKO) strategy in which the obligatory SHH co-receptor, Smoothened (SMO), is selectively deleted in GFAP-expressing cells. Mice carrying a floxed *Smo* allele (*Smo*^*fl*/*fl*^)^40^ were crossed with transgenic mice harboring Cre recombinase under control of the full length mouse *Gfap* promoter (line 73.12)^41^ to produce *GFAP-Cre*;*Smo*^*fl*/*fl*^ (Smo CKO) mice. In these mice, Cre-mediated recombination occurs in early postnatal GFAP-expressing astrocyte progenitor cells that produce most cortical astrocytes, enabling targeted deletion of SMO in most astrocytes^15^. SMO is required to transduce Shh signaling through both canonical, Gli1-dependent and non-canonical, Gli1-independent pathways^42^. We previously showed a dramatic reduction in the number of Gli1 cells in these mice, indicating effective deletion of SMO and loss of Shh activity^15^.

We performed stab injury in the forebrains of adult Smo CKO mice and wild type (WT) controls (*Smo*^*fl*/*fl*^, not carrying the Cre transgene) and evaluated the phenotypic properties of reactive astrocytes in Smo CKO mice at 7 dpi. We found that reactive astrocytes in Smo CKO mice exhibited pronounced changes in GFAP staining and morphology in a manner similar to that observed in WT mice. In both Smo CKO and WT tissues, upregulation of GFAP was most dramatic in astrocytes within the lesion area, which also showed unipolar or bipolar-like morphology. These cells displayed a highly-organized appearance, with elongated processes oriented in a parallel fashion towards the lesion and a dense thicket of GFAP-stained, overlapping processes in the center of the lesion (Supplementary Fig. S4). In contrast, astrocytes distal from the lesion in both Smo CKO and WT mice showed more moderate, but nevertheless pronounced, upregulation of GFAP compared to the contralateral hemisphere and retained a stellate morphology with non-overlapping processes that maintained territorial boundaries (Supplementary Fig. S4). Reactive astrocytes in the lesion area also showed pronounced cellular hypertrophy, with significant increases in cell volume compared to uninjured tissues in both Smo CKO mice and WT controls, though there was no difference between genotypes (Supplementary Fig. S4). Analysis of proliferation showed that in both Smo CKO animals and littermate controls, BrdU-labeled cells were observed primarily within the lesion area. Stereological estimation of the total number of BrdU cells within the lesion area showed a modest reduction in Smo CKO mice compared to WT controls (71124 ± 5989 and 92902 ± 10751, respectively), however this difference was not statistically significant (Supplementary Fig. S4).

Reactive astrogliosis and glial scar formation perform critical neuroprotective functions, including limiting the invasion of peripheral leukocytes, mitigating neuronal cell death^25,35^. To investigate the possibility that failure to restore Shh signaling impairs the resolution of injury at long term time points, we examined neuronal survival and inflammation at 28 dpi, when acute trauma, including inflammation and cell death, is largely resolved and lesions have matured^16^. We evaluated neuronal survival by immunostaining for the neuronal-specific protein, NeuN, and quantified the number of labeled cells in the lesion area. In uninjured brains, both Smo CKO mice and WT controls show no difference in the number of neurons (309,634 ± 13,043 and 300,142 ± 1942 cells respectively, 3 animals per genotype, Supplementary Fig. S5), suggesting that disrupting Shh signaling in astrocytes does not interfere with neuronal production or survival during development. Following injury, both WT and Smo CKO mice exhibited fewer neurons in the lesion area, suggesting injury-induced neuronal cell death. However the number of neurons was not significantly different between Smo CKO mice and WT controls (206,444 ± 4495 and 217,294 ± 34,650, respectively, 3 animals per genotype, Supplementary Fig. S5), suggesting that injury resolution proceeds along a time course similar to that in WT control tissues. In order to evaluate inflammation, we performed immunostaining for the glycoprotein, CD45, which is expressed by peripherally derived leukocytes. We observed dense localization of CD45 cells within the superficial layers of the cortex, at the entry point of the blade, that extended ventrally along the blade track (Supplementary Fig. S5). The relative abundance and distribution of CD45 cells was similar between Smo CKO mice and WT controls (Supplementary Fig. S5), suggesting effective resolution of inflammation. Reactive astrocytes surround and corral leukocytes, limiting their migration into parenchyma and mitigating inflammation^25^. Double immunofluorescence staining for GFAP and CD45 showed GFAP stained processes intermingled with CD45 cells at the lesion site in both Smo CKO and WT tissues (Supplementary Fig. S5), suggesting that loss of Shh signaling does not impair intercellular interactions between reactive astrocytes and leukocytes.

Finally, in order evaluate effective restoration of the BBB, we examined the persistence of blood serum proteins in parenchymal tissues. We performed immunostaining for mouse IgG in Smo CKO mice and controls at 14 and 28 dpi. At 14 dpi, the ipsilateral cortex of WT mice showed greater staining intensity in tissues at the lesion site, compared to the contralateral cortex, indicating the presence of serum proteins in parenchymal tissues surrounding the lesion (Supplementary Fig. S6). Smo CKO mice showed a similar staining pattern as that observed in WT controls, showing increased staining predominantly in tissues surrounding the lesion, and greater than that observed in the contralateral cortex (Supplementary Fig. S6). Notably, there was little to no staining in tissues beyond the lesion, even in the ipsilateral hemisphere, suggesting that disruption of the BBB is restricted to the lesion area, and that the BBB is not impaired in Smo CKO mice. Moreover, staining intensity at the lesion was comparable in Smo CKO mice to that in WT tissues. At 28 dpi, WT and Smo CKO tissues showed a marked reduction in serum proteins in the lesion area, compared to 14 dpi, suggesting effective resolution of the BBB by this time point (Supplementary Fig. S6). Staining intensity was comparable between ipsilateral and contralateral hemispheres, and between genotypes. These data suggest that failure to restore Shh signaling does not impair resolution of the BBB. Taken together, these data suggest that chronic interruption of Shh signaling selectively in astrocytes does not impede injury resolution, suggesting that astroglial scar production and maturation remain intact. Moreover, these data further support our observation that Shh activity is lost in reactive astrocytes.

### Shh signaling is sufficient to restrict leukocyte migration

**Figure 5:**
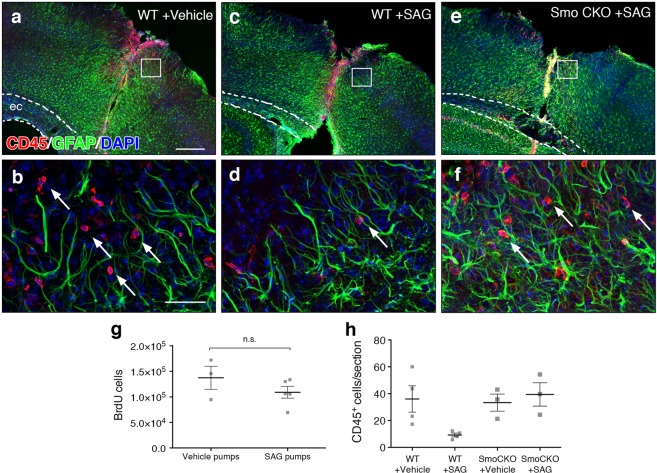
Activation of Hh signaling attenuates leukocyte migration. (**a**–**f**) Low power epifluorescent (**a**,**c**,**e**) and high power, confocal images (**b**,**d**,**f**) of immunofluroescent double staining for CD45 (red) and GFAP (green) in the lesion area of wildtype (**a**-**d**), or Smo CKO (**e**, **f**) mice that received vehicle (**a**,**b**) or SAG (**c**–**f**). Scale bar, 250 µm, (**a**,**c**,**e**); 50 µm, (**b**,**d**,**f**). Boxes in a, c, e depict regions analyzed in confocal images for CD45 quantification. Dotted lines indicate external capsule. (**g**) Stereological quantification of the estimated total number of BrdU cells in the lesion area. Data points represent individual animals, bars represent mean ± SEM. Statistical significance was assessed by Student’s t-test (*p* = 0.2569). (**h**) The number of CD45 labeled cells in parenchymal tissues adjacent to the lesion. Data points represent individual animals, bars represent mean ± SEM. Statistical significance was assessed by one-way ANOVA (*p* = 0.046), with post-hoc Tukey’s multiple comparisons test.

Following invasive injuries, reactive, scar forming astrocytes limit the spread of peripherally-derived leukocytes that gain access to the CNS, and play a critical role in repairing a damaged BBB^25,35^. Although there is a reduction in Shh signaling immediately after injury, the restoration of Shh activity at 14 dpi is correlated with injury resolution and BBB repair^18,35^. Shh signaling has been shown to promote BBB function and modulate immune response in healthy mice and in experimental autoimmune encephalitis (EAE), an animal model of multiple sclerosis^8^. We therefore examined whether pharmacological activation of the Shh pathway in astrocytes plays a role in mitigating the inflammatory environment of a stab injury. We implanted mini-osmotic pumps in wild type mice immediately after stab injury and infused the SMO agonist, SAG, or vehicle directly into the lesion site for 7 days. As in previous experiments, mice received daily injections of BrdU over days 3–6 after injury. Tissues were harvested for analysis on the last day of pump infusion. We performed stereological quantification of BrdU cells in the lesion area, and found no significant difference in proliferation between SAG and vehicle treated mice (137,416 ± 22,740 and 109,119 ± 11,485, in vehicle and SAG, respectively, Fig. 5). However, evaluation of leukocytes in injured tissues showed a pronounced reduction in SAG treated tissues compared to vehicle treated controls. A dense population of CD45 labeled cells were observed in the blade track, extending from the pial surface and throughout the depth of the cortex (Fig. 5). These cells were surrounded by the elongated processes of GFAP-expressing astrocytes. Although we detected a moderate reduction in the density of CD45 cells at the lesion site in SAG-treated tissues, some sections were torn at the site of cannula insertion, so it was not possible to reliably quantify CD45 cells at the lesion. We therefore quantified the number of CD45-labeled cells in tissues adjacent to the lesion site from high resolution confocal images. In SAG treated mice, there was a 74% reduction in the number of CD45-labeled cells compared to vehicle controls (36.04 ± 9.8 and 9.25 ± 1.4, in vehicle and SAG, respectively, Fig. 5), suggesting that infiltration of peripheral leukocytes is mitigated in response to pharmacological activation of the pathway. To determine whether SAG is acting on SMO in astrocytes or in other cell types, we implanted mini-pumps loaded with SAG or vehicle in Smo CKO mice. We reasoned that if SAG is acting on SMO in astrocytes, its deletion in these mice should abrogate the effect on leukocyte migration. Indeed, the number of CD45-labeled cells in Smo CKO mice that received SAG was not significantly different from those that received vehicle, and was comparable to vehicle treated WT mice (Fig. 5), suggesting that astrocytes are the key cellular effectors in SAG-mediated restriction of leukocyte invasion following invasive injury. These data further show that despite a reduction in availability of endogenous SHH, reactive astrocytes in the lesion area remain competent to respond to Shh signaling, and can mitigate inflammation upon exogenous activation of the Shh pathway. Although we cannot rule out the possibility that other cell types also express SMO, these data suggest that the anti-inflammatory effect of SAG is mediated by astrocytes. These data are in agreement with previous studies showing an anti-inflammatory role for SHH in injured tissues^8^.

## Discussion

In this study, we demonstrate that following an acute, focal insult, there is a pronounced loss of Shh signaling in reactive astrocytes in a temporally and spatially defined manner. We show that gene expression of *Shh* is diminished after injury, with a concomitant reduction in *Gli1* expression in astrocytes undergoing severe gliosis. Genetic labeling of cortical astrocytes expressing *Gli1* before injury shows these cells exhibit key features of reactive astrogliosis including upregulation of GFAP, cellular hypertrophy, and injury-induced proliferation in a manner similar to Gli1-negative astrocytes. Loss of Shh activity is most pronounced in tissues at the lesion site, whereas cells distal to the lesion continue to express *Gli1*, suggesting that Shh signaling is negatively regulated in cells experiencing the most severe damage but persists in healthier tissues distal to the injury. Loss of Shh activity occurs within 24 hours of the insult, and persists for up to two weeks. Interestingly, Shh signaling is restored to baseline levels two weeks after injury, as the glial scar reaches maturation, and tissues approach injury resolution^16^. Although endogenous Shh activity is downregulated after injury, activation of Shh signaling through exogenous application of the Smo agonist, SAG, is sufficient to limit parenchymal invasion of peripheral leukocytes by astrocytes, restricting inflammation in tissues adjacent to the primary lesion. Taken together, these data suggest that Shh signaling in astrocytes is dynamically regulated in a context-dependent manner. In addition, these data support a role for astrocytic Shh signaling in attenuating inflammation following an acute, focal injury.

It has been widely reported that SHH and GLI1 are upregulated in various CNS injury models, including spinal cord injury, cortical freeze, stab wound, and EAE^1,2,6–8^. Our findings stand in marked contrast to these reports. Using several parallel approaches, including direct and indirect transgenic reporter lines, absolute quantification of transcripts of multiple transcriptional targets of the Shh signaling pathway, as well as a conditional mutant approach in which we selectively abolished Shh signaling in astrocytes, we find no evidence of upregulation of Gli-dependent, canonical Shh signaling following injury. Moreover, these data argue against a glial source of SHH, as has been previously reported^1,7,8^. SHH is required for all transcriptional activation of *Gli1*. In studies of vertebrate early embryogenesis, where Shh signaling and its Gli transcriptional effectors are best understood, *Gli1* expression closely follows *Shh* expression, temporally and spatially in the developing embryo. Notably, in Shh mutants (*Shh*^*−*/*−*^), there is a failure to observe transcriptional activation of *Gli1*, demonstrating the absolute requirement for SHH in transcriptional activation of *Gli1* in the nervous system^13^. Using both a direct reporter approach and a genetic inducible recombination strategy, together with high resolution confocal microscopy and single cell analysis, we observed a 76% reduction in the number of Gli1 cells in the lesion area following injury. Our data reveal a pronounced loss of *Gli1* expression at the lesion site as early as 1 dpi, suggesting rapid loss of Shh activity. Reporter-positive cells were strikingly absent from the lesion area in both *Gli1*^*nlacZ*/+^ mice and in *Gli1*^*CreER*/+^;*R26*^*tdT*/*tdT*^ mice that received tamoxifen after injury, arguing against upregulation of *Gli1* in other cell types. Notably, tamoxifen administered to intact mice before injury showed an abundance of marked cells in the lesion area, arguing against cell death of Gli1 astrocytes in response to injury. In addition, two transcriptional targets of Shh signaling, *Gli1* and *Ptc*, show no evidence of transcriptional activation, as measured by direct quantification of transcript copy number from lesioned tissues. Consistent with this, quantification of *Shh* transcripts from the lesion area similarly showed a reduction in copy number, suggesting that loss of *Gli1* results from a loss of available SHH protein. Finally, abolishing Shh signaling in all astrocytes using the constitutive GFAP-Cre driver, has no effect on many key phenotypic and physiological hallmarks of reactive astrogliosis, or on neuronal survival, further arguing against an upregulation of Shh signaling after injury. Taken together these data demonstrate a reduction in canonical, Gli-dependent Shh signaling following injury.

Despite widespread reports that Shh signaling increases following injury, studies demonstrating injury-induced reduction in Shh are increasingly emerging. *Shh* and *Gli1* expression are diminished following traumatic brain injury and demyelination^43–45^. In the adult subventricular zone (SVZ), inhibition of *Gli1* in adult neural stem cells improves remyelination^46^. Interestingly, *Shh* and *Gli1* are also downregulated in lung tissues after injury, during active proliferation of mesenchymal cells as tissues undergo remodeling^47^. Both *Shh* and *Gli1* recover to baseline levels upon restoration of cellular quiescence and the resolution of injury^47^. These studies demonstrate that injury-induced loss of Shh signaling occurs in different injury models, and in various tissues, following acute trauma, and suggests that this may be a general feature of Shh signaling. Notably, these studies share the same molecular genetic strategies to detect and manipulate Shh signaling as those used in this study. These transgenic mouse models are well-established and have long been used in studies of the developing nervous system^12,40^. They provide sensitive and reliable readouts of Shh activity *in vivo*. Further studies are needed to resolve the apparently conflicting reports on acute levels of Shh activity after injury.

Interestingly, despite our observation that Shh signaling is downregulated after injury, expression of *Ptc* and *Smo* remains intact, suggesting that astrocytes remain capable of transducing Shh signal. This is in agreement with previous reports demonstrating that exogenous activation of the pathway, through application of Shh or other agonists, stimulates proliferation of neural progenitor cells, increases neurite outgrowth, and restores BBB integrity^9,48,49^. In addition, reactive astrocytes adopt neural stem cell properties *in vitro* in a Shh-dependent manner when isolated from invasive injuries, such as the stab wound model used here^7^. Thus, although endogenous Shh signaling is downregulated after injury, pharmacological stimulation of the pathway is sufficient to promote cellular programs that are associated with neural repair. Indeed, our observation that SAG administration dramatically limits invasion of peripheral immune cells into parenchymal tissues suggests Shh signaling mitigates inflammation, consistent with previous reports^8^. The effect of SAG is SMO-dependent and is mediated by astrocytes, as genetic deletion of SMO selectively in astrocytes fails to produce the same effect. While the precise mechanism by which SMO activation in astrocytes mediates interactions with leukocytes remains to be identified, this suggests that Shh activity stimulates cellular programs that promote neuroprotection.

Shh signaling has a well-established role in regulating proliferation of neural precursor cells in the developing and adult CNS^31–34^. Although astrocytes in the healthy CNS do not undergo constitutive proliferation, severe focal trauma, such as spinal cord injury or the stab injury model used here, triggers robust proliferation of reactive astrocytes that, together with fibroblasts and other cells, comprise the glial scar^10,16^. Our previous study identifying astrocytes as the predominant cells expressing *Gli1* outside the neurogenic niches in the adult CNS, together with previous reports describing an upregulation of SHH and GLI1, suggested that SHH may regulate proliferation of scar forming reactive astrocytes, and subsequently impair astrocytic contribution to astroglial scar production. Our unexpected observation that Shh signaling is lost in reactive astrocytes adjacent to the lesion instead suggests that Shh activity is negatively regulated in cells undergoing active proliferation, and suggests that astroglial scar formation is independent of Shh signaling. Consistent with this, reactive astrocytes in Smo CKO mice exhibit phenotypic properties indistinguishable from WT controls. Notably, we also did not detect a difference in neuronal survival or resolution of the BBB in Smo CKO mice compared to WT controls, suggesting that key functional outcomes of astroglial scar production persist, despite permanent interruption of Shh signaling in astrocytes. Together with the observation that injured tissues experience a loss in Shh signaling during the initiation of an astroglial scar, these data suggest that astroglial scar production is independent of astrocytic Shh signaling.

The loss of Shh signaling in cells acutely undergoing severe activation suggests that SHH may regulate gene expression programs that repress the reactive state^50^. Indeed, our previous study demonstrated that Smo CKO mice exhibit an upregulation of GFAP and mild hypertrophy in cortical astrocytes, in the absence of an injury^15^. However these cellular phenotypes were not observed in all cortical astrocytes, despite the widespread loss of SMO in these cells^15^. Moreover, Gli1 astrocytes in the cortex are intermingled with Gli1-negative astrocytes, which do not exhibit astrocyte activation in the intact brain, arguing against a role for Shh signaling in repressing a reactive phenotype. Alternatively, the acute and highly inflammatory environment that develops after disrupting the BBB may actively repress Shh activity, perhaps in response to signaling from blood-borne macrophages that have gained entry into the nervous system. Indeed, loss of Shh activity is correlated with highly inflammatory environments. These results reveal context-dependent regulation of Shh signaling in astrocytes and suggest that differential gene expression programs between healthy and severely reactive astrocytes may, in part, be mediated by Shh signaling.

## Materials and Methods

### Animals

All experimental protocols were approved by the Drexel University Institute for Animal Care and Use Committee, and experiments were conducted in accordance with approved protocols. Male and female adult animals between 3 and 6 months of age were used for this study. The following transgenic mouse lines were used: *Gli1*^*nLacZ*/+^ ^13^, *Gli1*^*CreER*/+^ ^12^, *R26*^*tdTom*/*tdTom*^ ^19^, *mGFAP-Cre*^41^, and *Smo*^*fl*/*fl*^ ^40^. Animals were maintained on a 12 h light/dark cycle and given access to food and water ad libitum.

### Tamoxifen

Tamoxifen (Sigma, T5648-1G) was diluted to a final concentration of 20 mg/ml in corn oil. Adult *Gli1*^*CreER*/+^;*R26*^*tdTom*/*tdTom*^ mice received 250 mg/kg of tamoxifen by oral gavage for 1 or 3 days consecutively, and tissue was harvested 2 weeks later, unless otherwise noted.

### Forebrain Stab Injury

Mice were subjected to forebrain stab injury as previously described^17,18^. Briefly, a 3 mm × 3 mm craniotomy was performed 1 mm caudal and lateral to bregma in the right parietal bone. A sterile no. 11 scalpel blade was stereotaxically inserted 3 mm into the brain, and moved to the rostral and caudal edges of the craniotomy three times creating a longitudinal lesion. The blade was then slowly removed, and the skin was sutured. Animals received post-operative injections of carprofen, s.c. (Rimadyl, 6.8 mg/kg, MedVet, RXRIM-INJ), and buprenorphine i.p. (0.05-0.1 mg/kg, MedVet, RXBUPRENOR5) for 2 additional days following surgery.

### BrdU

BrdU (Sigma, B9285-1G) was dissolved in 0.007 N NaOH and administered via i.p. injection. For long term experiments, mice received daily injections of 200 mg/kg BrdU over days 3–6 after injury, and were perfused at 7, 14, or 28 dpi. For uninjured or short term (1 and 3 day survival) experiments, mice received a single injection of 200 mg/kg BrdU 2 hours before perfusion.

### Perfusion and histology

Animals were given an i.p. injection of Ketamine/Xylazene/Acepromazine cocktail and transcardially perfused with 20 ml of 10 mM PBS followed by 60 ml of 4% paraformaldehyde solution. Brains were dissected and post fixed in 4% paraformaldehyde for 4–6 hours followed by cryoprotection in 30% sucrose and stored at 4 °C for at least 48 hours or until ready for sectioning. Serial sections were collected on a cryostat at 40 μm and stored at 4 °C in TBS with 0.05% sodium azide. Immunohistochemistry was performed on free floating tissues on every 12^th^ section using the following primary antibodies for fluorescence: rabbit anti-βgal (1:1k, MP Bio), chicken anti-βgal (1:1k, Abcam), rabbit anti-GFAP (1:1k, DAKO), mouse anti-NeuN (1:1k, Millipore), rabbit anti-RFP (1:500, MBL), rat anti-CD45 (1:200, BDBiosciences), rabbit anti-S100β (1:1k, DAKO), rabbit anti NG2 (1:500, Millipore) and sheep anti-BrdU (1:500, Maine Biotechnology Services). For BrdU staining, tissue was preincubated in 2 N HCl for 30 minutes and neutralized with 0.1 M TBS before incubation in block and primary antibody. Fluorescence labeling was achieved using species-specific AlexaFluor-tagged secondary antibodies, Alexa488, Alexa568, or Alexa647 (Life Technologies), followed by counterstaining with DAPI (Life Technologies). In some instances, S100β and GFAP were used in cocktail, with the same AlexaFluor-tagged secondary antibody wavelength, to definitively label astrocytes. For brightfield immunostaining, tissues were quenched in TBS with 0.3% H_2_O_2_ and 30% methanol for 30 minutes prior to incubation in block and primary antibody. The following antibodies were used: rabbit anti-S100β (1:40k, DAKO), rabbit anti-βgal (1:40k, MP Bio), rabbit anti-GFAP (1:20k, DAKO), rabbit anti-RFP (1:5k, MBL), rat anti-CD45 (1:4k, BDBiosciences), mouse anti-NeuN (1:5k, Millipore), and sheep anti-BrdU (1:2k, Maine Biotechnology Services). For brightfield staining, species-specific biotinylated secondary antibodies (Vector) were used at 1:400 followed by incubation in avidin-biotin complex (ABC, Vector). For Mouse IgG immunostaining, tissues were first incubated in block, then goat anti mouse secondary antibody (Vector, 1:2k) for 1 hour each prior to ABC incubation. Visualization was achieved using 3’−3 diaminobezedine (DAB, Vector) as the developing agent.

### Microscopy

Stained sections were examined and imaged in brightfield and fluorescence using an upright microscope (Zeiss) and Stereo Investigator software (MBF Bioscience). Confocal images were obtained on an inverted microscope (Zeiss) using Axiovision software. Single-cell analysis of co-labeling was evaluated on double-stained immunofluorescent tissues from confocal z-stacks collected with 20×, 40× or 63× oil objective, with a 1 µm slice distance. Images were collected from at least 3 sites located throughout the lesion area, defined as 500 µm mediolaterally from the lesion, and from 4–5 sections across the rostrocaudal extent of the lesion or from equivalent levels in uninjured/contralateral sections from at least 3 brains. Cells were manually analyzed in ImageJ, using the Cell Counter Plugin to mark individual cells.

### Stereological analyses

The total number of cells was estimated in tissues stained for brightfield microscopy. The ipsilateral cortex of injured brains was analyzed in a series of 4–5 sections, spaced 480 µm apart, encompassing the lesion. We used a modified optical fractionator and stereological image analysis software (Stereo Investigator, MBF Bioscience) operating a computer-driven stage attached to an upright microscope (Zeiss). The cortical area to be analyzed was outlined at low magnification, and counting frames were selected at random by the image analysis software. Cells were counted using a 40x objective and DIC optics. A target cell count of 300 cells was used to define scan grid and counting frame sizes, with a 2 μm guard zone. For all analyses, only cells with a clear and distinct labeled cell body were analyzed. Estimated cell body volumes were determined in RFP- or S100β-labeled cells using a nucleator probe, with 4 isotropic uniform random (IUR) rays emanating from the nucleus (StereoInvestigator, MBF Bioscience).

### Absolute quantification of RNA

The cortex was isolated, and a 3 mm × 3 mm region of tissue encompassing the lesion and lesion area was microdissected, removing underlying white matter. Control tissues included the intact contralateral hemisphere or uninjured brains, and were pooled. Total RNA was extracted using Trizol Reagent (Life Technologies, cat #15596026) and residual genomic DNA removed using the TURBO DNA-free kit (Ambion, Cat #AM1907), according to manufacturer’s instructions. RNA concentration and integrity were measured using a bioanalyzer, and RNA 6000 Nano Kit (Agilent, Cat #5067-1511). cDNA was synthesized using Applied Biosystems High-Capacity cDNA Reverse Transcription Kit (ThermoFisher, Cat# 4368814); 1ug of total RNA in a final reaction volume of 20ul reaction was converted to cDNA for each sample.

Absolute quantification of target transcripts was determined by Droplet Digital^TM^ PCR, using QX200 ddPCR EvaGreen Supermix (Bio-Rad, Cat# 1864034) according to manufacturer’s instructions. The primers used were as follows: *Gli1* (Fwd: 5′-GACGGAGGTCTCTTTGTCCG-3′; Rev: 5′- AACATGGCGTCTCAGGGAAG-3′), *Shh* (Fwd: 5′-TTTGGAAAGAGGCGGCACCC-3′; Rev: 5′-TGCACCTCTGAGTCATCAGCCG-3′), *Gfap* (Fwd: 5′-TTGCTGGAGGGCGAAGAAAA-3′; Rev: 5′-CTGGTGAGCCTGTATTGGGA-3′), *Ptc1* (Fwd: 5′-CTGGAGCAGATTTCCAAGGGGA-3′; Rev: 5′-TAATCCCACAGCGAAGGCCC-3′), and *Smo* (Fwd: 5′-CCTGAAGGCTGCCCAAACGA-3′; Rev: 5′-TTCGCACCAAGGGTGCTTCA-3′). Absolute quantification of each transcript was calculated using the Quantasoft Version 1.7 software (Bio-Rad) after droplets were run through the QX200 Droplet Reader (Bio-Rad), and are represented as copies of transcript per microliter of amplified PCR mixture. All samples were run in triplicate, and a no template control for each reverse transcription reaction was included in each assay.

### Miniosmotic pump implantation

Miniosmotic pumps (Alzet 1007D) were assembled under sterile conditions, filled with 1uM Smoothened agonist (SAG, Millipore) dissolved in 0.9% saline or vehicle, and allowed to equilibrate overnight at 37 °C. Pump installation was performed immediately following a modified 3 mm long x 2 mm wide forebrain stab injury. The cannula of the brain infusion kit (Alzet Brain Infusion Kit 3) was inserted directly into the lesioned cortex at a depth of 2 mm from the skull. Animals were given the same post-operational Carprofen, Buprenorphine, and BrdU injections as detailed above for stab injury. Analysis was performed from confocal z stacks collected at 20×, from multiple sites within the lesion area, defined as tissues within 500 µm mediolaterally from the lesion. Cells were analyzed from one site per section, from 3 sections most proximal to the lesion. Tissues were analyzed from at least 3 animals per group, and are reported as the mean number of CD45 cells/section from each of 3–4 animals.

### Statistics

All statistical analyses were performed using Prism 6 (GraphPad).

## Supplementary information

Supplementary Dataset

## Data Availability

The datasets generated and/or analyzed during the current study are available from the corresponding author on reasonable request.
