# Supplementary material for: Sonic hedgehog signaling is negatively regulated in reactive astrocytes after forebrain stab injury
Source: Sci Rep. 2019 Jan 24;9:565. doi: 10.1038/s41598-018-37555-x (PMC6345977; doi:10.1038/s41598-018-37555-x)
Supplement: Supplementary file 1 — Supplementary Dataset [file 41598_2018_37555_MOESM1_ESM.pdf]

TITLE: Sonic hedgehog signaling is negatively regulated in reactive astrocytes after forebrain stab injury

AUTHORS: R. Vivian Allahyari[1], K. Lyles Clark[1,2], Katherine A. Shepard[1], A. Denise R. Garcia[1,*]
[1]Departments of Biology and Neurobiology and Anatomy, Drexel University, Philadelphia, PA, 19104, USA
[2]Current address, Mahoney Institute for Neurosciences, Perelman School of Medicine, University of Pennsylvania, Philadelphia, Pennsylvania; and Department of Anesthesiology and Critical Care Medicine, Children's Hospital of Philadelphia, Philadelphia, Pennsylvania.

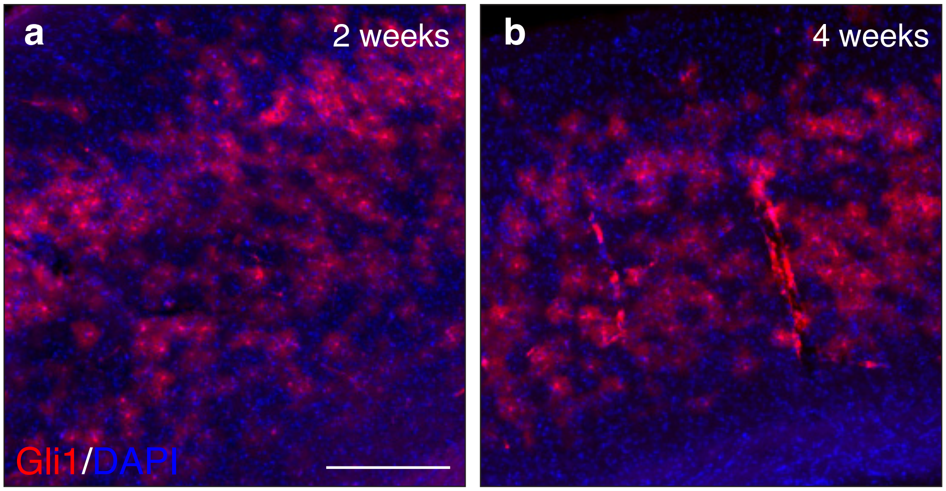

Supplementary Figure S1. Number and distribution of marked cells in *Gli1*$^{CreER/+}$*;R26*$^{tdTom/tdTom}$ mice is similar at 2 and 4 weeks after tamoxifen. Tomato expression (red) in the cortex of an adult *Gli1*$^{CreER/+}$*;R26*$^{tdTom/tdTom}$ mouse that received tamoxifen two (**a**) and four (**b**) weeks earlier. Tissues counterstained with DAPI (blue). Scale bar, 250μm.

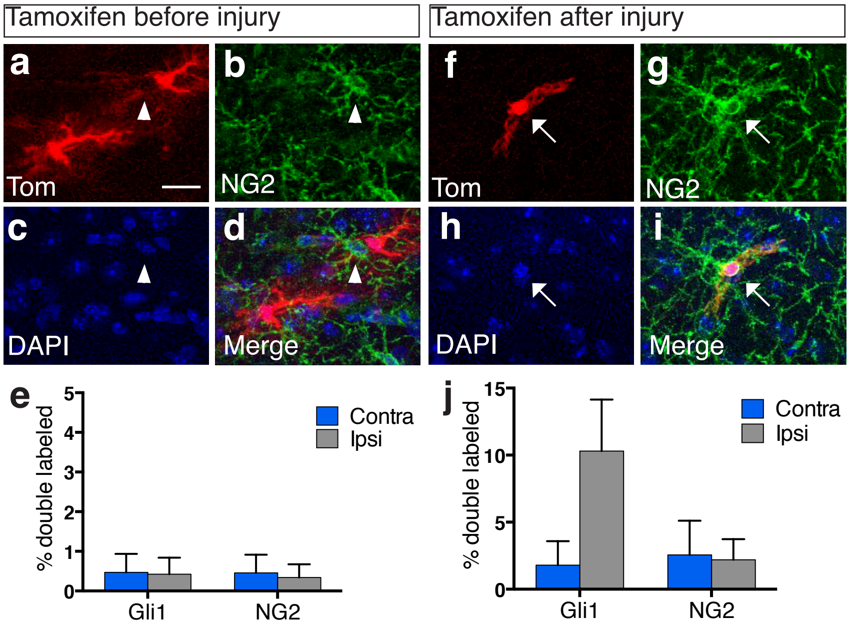

Supplementary Figure S2. NG2 cells do not express Gli1. (a-d, f-i) Maximum projection images from confocal z-stacks from tissue adjacent to the lesion, showing marked Gli1 and NG2 labeled cells. Tomato (red), and NG2 (green) in the ipsilateral cortex of an injured, adult $Gli1^{CreER/+}$; $R26^{tdTom/tdTom}$ mouse that received tamoxifen before (a-d), or after (f-i) injury. Arrow head in **d** shows NG2 cell. Arrow in **i** shows double labeled cell. Note the pericytic morphology, in contrast to the astrocytic morphology of marked cells in (a). Tissues counterstained with DAPI (blue, **c, h**). Scale bar, 25 μm. **(e)** Single cell analysis of the proportion of Gli1 cells and NG2 cells that are double labeled from animals marked before injury in the contralateral (n = 525 Gli1 and n = 505 NG2 cells analyzed from 2 animals) and ipsilateral hemispheres (n = 703 Gli1 and 785 NG2 cells analyzed from 2 animals). **(j)** Single cell analysis of the proportion of Gli1 cells and NG2 cells that are double labeled from animals marked after injury in the contralateral (n = 467 Gli1 and n = 523 NG2 cells analyzed from 2 animals) and ipsilateral hemispheres (n = 130 Gli1 and 679 NG2 cells analyzed from 2 animals). Note that there are far fewer $Gli1^{+}$ cells in the injured cortex when animals are marked after injury.

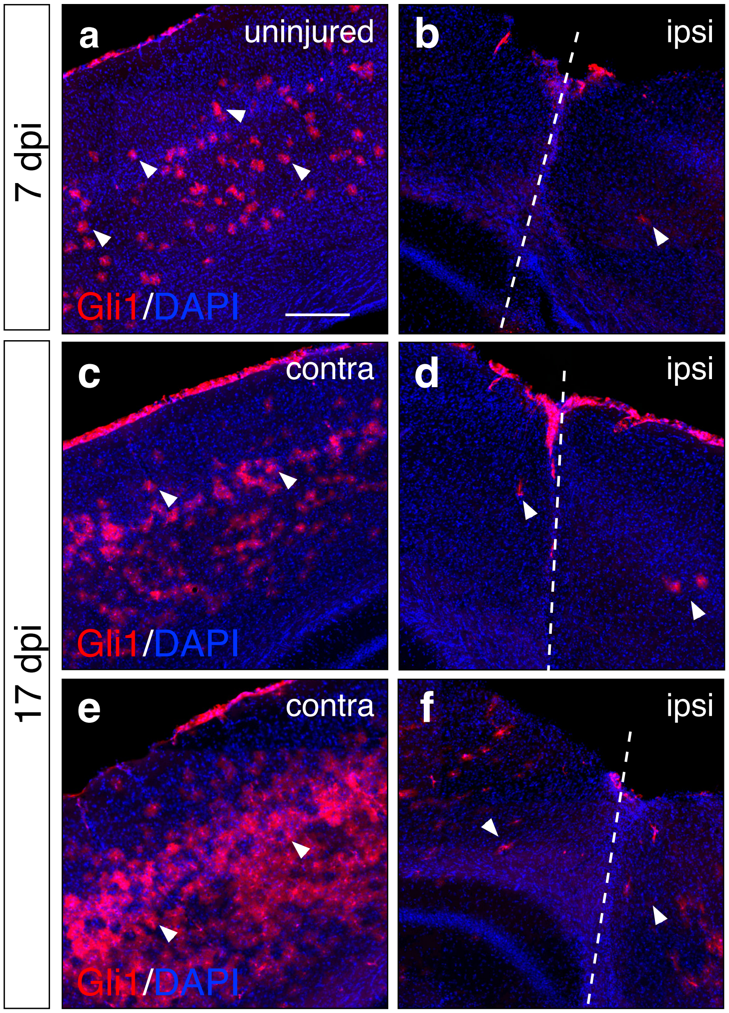

Supplementary Figure S3. Loss of Gli1 expression after injury. (a) Immunohistochemistry (IHC) for RFP labeling tdtomato expression (red) in the cortex of an uninjured, adult *Gli1*$^{CreER/+}$*; R26*$^{tdTom/tdTom}$ mouse that received a single dose of tamoxifen, two weeks earlier. (**b-d**) IHC for RFP (red) in the ipsilateral (b, d) and contralateral (c) cortex of an adult *Gli1*$^{CreER/+}$*;R26*$^{tdTom/tdTom}$ mouse that received a single dose of tamoxifen at 3 dpi. **(e, f)** IHC for RFP (red) in the ipsilateral (f) and contralateral (e) cortex of an adult *Gli1*$^{CreER/+}$*;R26*$^{tdTom/tdTom}$ mouse that received tamoxifen on days 3, 4, and 5 after injury. Tissues were harvested 7 (b) or 14 (c-f) days after the final dose of tamoxifen. Dashed line in b, d, and f depict blade track. Tissues counterstained with DAPI (blue). Scale bar, 250µm.

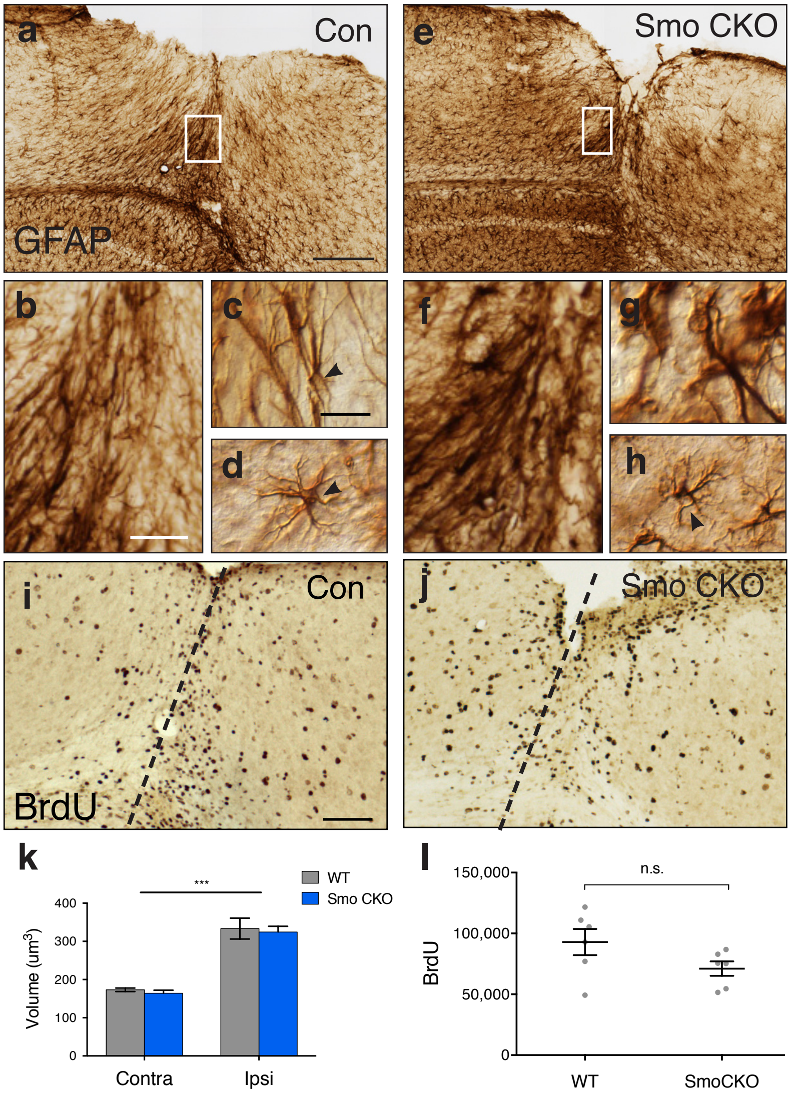

Supplementary Figure S4. Key hallmarks of reactive gliosis are not impaired in Smo CKO mice. (**a-h**) Brightfield immunohistochemistry for GFAP in control (a-d) and Smo CKO (e-h) mice 7 days after injury. Panels in b and f show enlarged photos of boxes in a and e depicting the thick, interdigitating GFAP-stained processes of astrocytes at the blade track. Scale bar, 250 μm. (c, g) Individual astrocytes with unipolar or bipolar morphologies, distinct from cells distal from the lesion (d, h) that maintain stellate morphologies, but nevertheless exhibit dramatic hypertrophy and upregulation of GFAP. Scale bar, 25 μm. (**i-j**) Brightfield immunostaining for BrdU in the lesion area of a WT control (i) and Smo CKO (j) mouse. Dotted line depicts the lesion site. Scale bar, 100 μm. (**k**) Stereological analysis of cell volulme of reactive astrocytes in the contralateral and ipsilateral hemispheres of of wild type and Smo CKO mice. Statistical significance was assessed by 2-way ANOVA with post-hoc Tukey's multiple comparisons test ($n > 150$ cells from each hemisphere of 2 WT and 3 CKO animals): hemisphere ($F_{1,5} = 92.41$, ***$p = 0.0002$), genotype ($F_{1,5} = 0.287$, $p = 0.615$, n.s.). No interaction was found between genotype and hemisphere ($F_{1,5} = 0.296$, $p = 0.600$, n.s.). (**l**) Estimated total number of BrdU cells in the lesion area at 7 dpi. Data points represent individual animals. Data represent mean ± SEM. Statistical significance was assessed by unpaired Student's t-test ($p = 0.1072$).

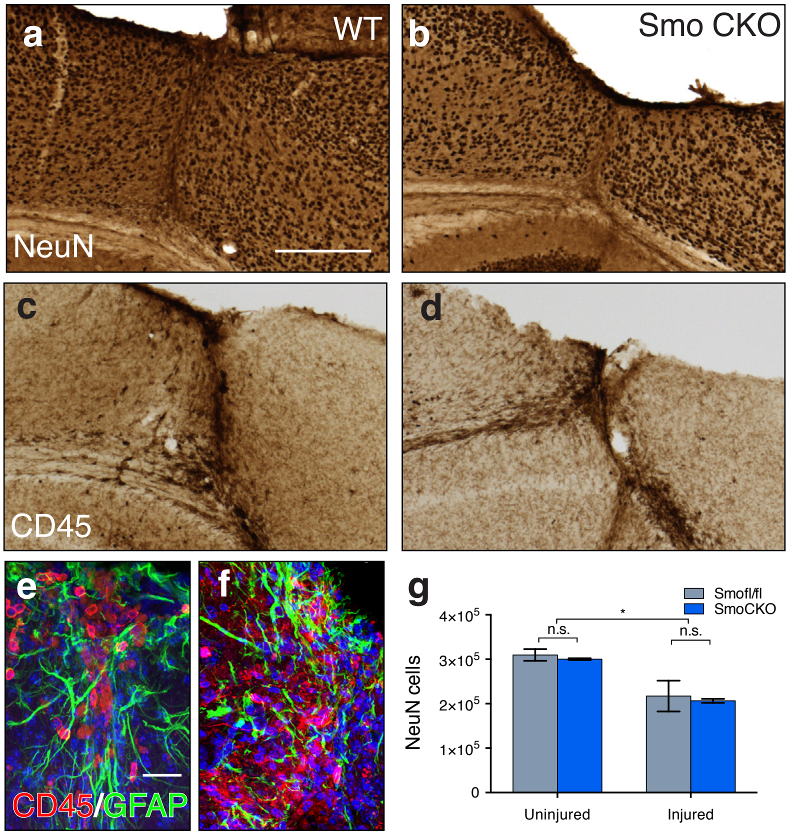

Supplementary Figure S5. Injury resolution is unimpaired in Smo CKO mice. (**a-d**) Brightfield immunohistochemistry for NeuN (a-b) and CD45 (c-d) in wild type (a, c) and Smo CKO (b, d) mice 28 dpi. Scale bar, 250 μm. (**e-f**) Immunofluorescent double labeling for GFAP (green) and CD45 (red) at the lesion site in wild type (e) and Smo CKO (f) tissue. Tissues counterstained with DAPI (blue). Scale bar, 25 μm. (**g**) Stereological estimation of the total numbers of neurons in the lesion area in wild type and Smo CKO tissues at 28 dpi (*n* = 3 animals per group, per genotype). Data represent mean ± SEM. Statistical significance was assessed by two-way ANOVA with post-hoc Tukey's multiple comparisons test: genotypes ($F_{1,8} = 0.296$, $p = 0.600$, n.s.), injury ($F_{1,8} = 24.81$, $^{**}p = 0.001$). No interaction was found between genotype and injury ($F_{1,8} = 0.001$, $p = 0.971$, n.s.).

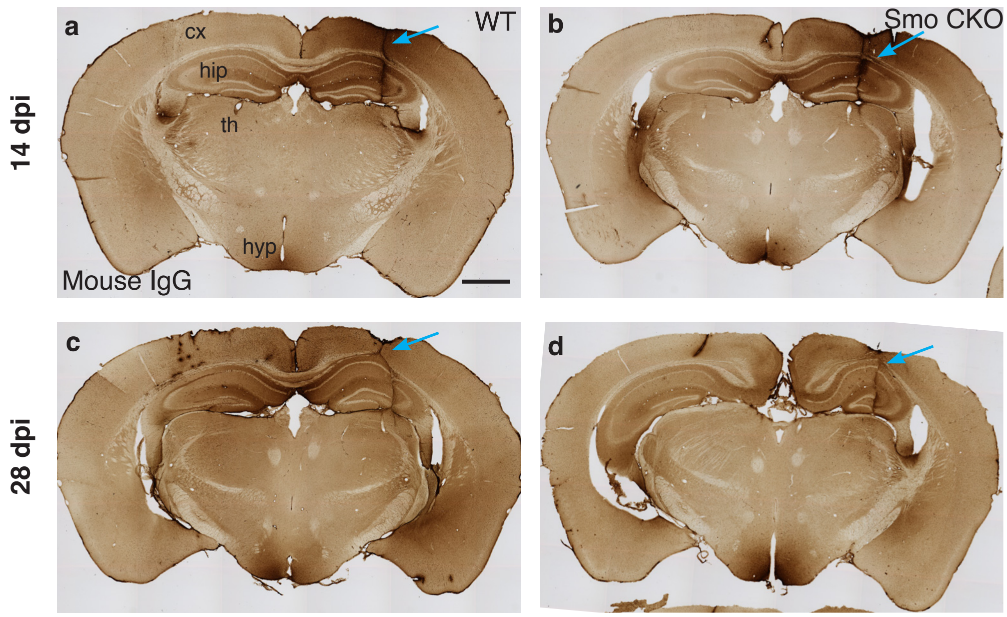

Supplementary Figure S6. Loss of Shh signaling in astrocytes does not prolong blood protein leakage after injury. (**a-d**) Brightfield immunohistochemistry for mouse IgG in control (a, c) and Smo CKO (b, d) mice at 14 (a, b) and 28 (c, d) days after injury. Arrow indicates lesion. Cortex (cx), hippocampus (hip), thalamus (th), and hypothalamus (hyp) are labeled. Scale bar, 1 mm.
